# The Molecular Mechanism Underlying Pro-apoptotic Role of Hemocytes Specific Transcriptional Factor Lhx9 in *Crassostrea hongkongensis*

**DOI:** 10.3389/fphys.2018.00612

**Published:** 2018-05-28

**Authors:** Yingli Zhou, Fan Mao, Zhiying He, Jun Li, Yuehuan Zhang, Zhiming Xiang, Shu Xiao, Haitao Ma, Yang Zhang, Ziniu Yu

**Affiliations:** ^1^CAS Key Laboratory of Tropical Marine Bio-resources and Ecology, Guangdong Provincial Key Laboratory of Applied Marine Biology, South China Sea Institute of Oceanology, Chinese Academy of Sciences, Guangzhou, China; ^2^University of Chinese Academy of Sciences, Beijing, China

**Keywords:** *Crassostrea hongkongensis*, hemocytes, apoptosis, *Ch*Lhx9, *Ch*ASPP1, RNAi, RNA-seq

## Abstract

Hemocytes are the central organ of immune defense against pathogens by means of inflammation, phagocytosis, and encapsulation in mollusks. The well-functioning of the host immune system relies on the hemocytes’ task exertion and frequent renewal, but the underlying renewal mechanism remains elusive at the gene level. Here, we identified one transcription factor, LIM homeobox 9, in *Crassostrea hongkongensis* (*Ch*Lhx9) that could be involved in hemocyte apoptosis or renewal. *Ch*Lhx9 contains a homeodomain and two LIM domains. The expression profile of *Ch*Lhx9 showed that it was specific and had high expression in hemocytes, and it significantly increased under the bacterial challenge. RNA interference of *Ch*Lhx9 dramatically decreased the apoptosis rate of hemocytes when compared with a control group, which strongly implies its pro-apoptotic role in hemocytes. Furthermore, the genomic responses to the knockdown of *Ch*Lhx9 were examined through RNA-seq, which showed that multiple pathways associated with cell apoptosis, including the apoptosis pathway, hippo signal pathway and p53 signaling pathway, were significantly down-regulated. Meanwhile, seven of the key apoptotic genes were confirmed to be upregulated by *Ch*Lhx9, among which *Ch*ASPP1 (apoptosis stimulating protein of p53) was confirmed to induce hemocyte apoptosis strongly, which demonstrates that *Ch*ASPP1 was a downstream target mediated by *Ch*Lhx9 that caused apoptosis. In conclusion, tissue-specific transcription factor *Ch*Lhx9 induces hemocyte apoptosis through activating apoptotic genes or pathways, which could contribute to hemocyte renewal and immune defense in oysters.

## Introduction

Hemocytes function in both cellular and humoral defenses, which is the main means for oysters to cope with infectious agents (Chu, 1988; Feng, 1988; Lavine and Strand, 2002; Arefin et al., 2015). Eliminating pathogens relies on the ability of hemocytes to recognize foreign targets and induce apoptotic cell death (Lavine and Strand, 2002; Sokolova, 2009). In addition, activation-induced hemocyte death, as the second safeguard against autoimmunity, prevents an excessive immune response (Feig and Peter, 2007). The apoptosis of hemocytes plays an essential role in homeostasis of the immune responses to ensure well-functioning of the oyster immune system in resisting foreign microbes (Opferman and Korsmeyer, 2003; Hildeman et al., 2007; Hughes et al., 2010; Ghosh et al., 2015). Therefore, the mechanism that underlies oyster hemocyte apoptosis has drawn a large amount of attention and studies to obtain a better understanding of oyster autoimmunity and disease management.

Homeobox genes, which encode homeodomain transcription factors, are involved in the regulation of apoptosis, cellular differentiation and developmental processes in many metazoans (Mcginnis et al., 1984; Lohmann et al., 2002; Pearson et al., 2005; Kocak et al., 2013). E2A-PBX1, a chimeric homeobox protein, paradoxically induces both thymocyte death and lymphoma in E2A-PBX1 transgenic mice (Dedera et al., 1993). During the development of *Drosophila*, homeobox genes Dfd and Abd-B induce localized apoptosis to maintain normal segment boundaries, and Dfd directly binds to the enhancer of the downstream cell death promoting gene reaper (White et al., 1994; Lohmann et al., 2002). Recently, more studies have revealed that Hox genes, a subset of the homeobox genes, play a pivotal role in cell apoptosis for vertebrates (Raman et al., 2000). HOX-C9 activates the intrinsic pathway of apoptosis to inhibit the growth of neuroblastoma (Kocak et al., 2013) and the overexpression of HOXB1 to induce the death of HL60 leukemic cells by upregulating caspase2 and downregulating MDM2 (Petrini et al., 2013).

Recently, our previous studies showed that one homeobox gene, Lhx9, was specific and highly expressed in hemocytes in Hong Kong oyster, *C. hongkongensis* (Tong et al., 2015), an economically important and filter-feeding species in the coastal waters of the South China Sea (Volety et al., 1999; Lam and Morton, 2003). Lhx9 contains two specialized cysteine-rich LIM domains that are involved in protein-binding interactions and a homeodomain that is involved in DNA-binding interactions and the transcription of downstream genes (Sanchezgarcia and Rabbitts, 1994; Dawid et al., 1995; Agulnick et al., 1996). In mammals, Lhx9 is important for the development of gonads and is responsible for the invasion of the epithelium into the mesenchyme (Birk et al., 2000). In invertebrate, a homologous gene of Lhx9 is expressed in accessory cells to control the early regulation of wing development (Bourgouin et al., 1992).

However, compared to studies in model organisms, the function of Lhx9 in mollusks is still unknown. Therefore, in this study, we cloned and functionally studied a molluscan Lhx9 homolog from *C. hongkongensis*. *Ch*Lhx9 knockdown oysters harbor a significantly lower apoptosis rate compared to control oysters. RNA-sequencing analysis of *Ch*Lhx9 knockdown oysters indicated that it participates in the regulation of several cellular process pathways that are associated with cell apoptosis. Importantly, we identified seven crucial apoptosis genes that are regulated by *Ch*Lhx9 and revealed that *Ch*ASPP1, the downstream gene of *Ch*Lhx9, induces the apoptosis of hemocytes. Thus, *Ch*Lhx9 serves as a tissue-specific transcription factor to induce the apoptosis of hemocytes by activating the expression of apoptotic genes in *C. hongkongensis*.

## Materials and Methods

### Animals and Sample Collection

Healthy oysters (*C. hongkongensis*), which averaged 108 mm in shell height, were collected from Zhanjiang, Guangdong province, China, and kept in aerated seawater (18‰ salinity) at 25°C and fed with 0.8% *Tetraselmis suecica* and *Isochrysis galbana* for 1 week before processing. Different tissues (hemocytes, labial palps, heart, digestive gland, mantle, muscle, gonad, and gill tissues) were collected from at least three independent oysters for RNA extraction and flow cytometry. The collected samples were immersed in TRIzol, quickly frozen in liquid nitrogen, and then stored at -80°C until RNA isolation.

### Pathogen Challenge and *in Vivo* RNA Interference (RNAi)

For the pathogen challenge, the oysters were randomly divided into three groups, the *Vibrio alginolyticus*, the *Staphylococcus haemolyticus* challenge groups and the PBS control group, with each group in separate tanks. Oysters in the two challenged groups were injected with 100 μL *V. alginolyticus* or *S. haemolyticus* (suspended in 0.1 M PBS at a concentration of 1.0 × 10^9^ cells/mL) into the adductor muscle; oysters in the control group were injected with an equal volume of PBS. After treatment, the oysters were returned to the water tanks, and three individuals were randomly sampled at 3, 6, 12, 24, 48, and 72 h post-injection.

An *in vivo* dsRNAi experiment was conducted according to the previous reports (Liu et al., 2017). The oysters were divided randomly into two groups. In the experimental group, 100 μL of *Ch*Lhx9 dsRNA or *Ch*ASPP1 dsRNA (1 mg/μL) was injected into the adductor of each oyster. The oysters that received an injection of 100 μL EGFP dsRNA (1 mg/μL) were used as the control group. Hemocytes were sampled at 72 h post-injection. Then, 48 h after the knockdown of *Ch*Lhx9, the remaining oysters in the two groups received an injection of 100 μL of *V. alginolyticus* (suspended in 0.1 M PBS at a concentration of 1.0 × 10^9^ cells/mL) in the adductor muscle. The hemocytes were harvested at 24 h post-challenge.

### Cloning of *Ch*Lhx9 cDNA and Sequence Analysis

According to the sequence of *C. gigas* Lhx9 (GenBank accession number: XP011419207.1), gene-specific primers (**Supplementary Table S1**) were designed and used to clone the cDNA of *Ch*Lhx9. The primer pairs *Ch*Lhx9-F1/R1, TakaraUPM/*Ch*Lhx9-R2 and *Ch*Lhx9-F2/TakaraUPM were used to amplify the ORF of *Ch*Lhx9, 5′UTR of *Ch*Lhx9 and 3′UTR of *Ch*Lhx9. The synthesis of cDNA templates and a PCR program were used according to the SMARTer^®^ RACE 5′/3′ Kit (TaKaRa, Japan) recommendation. All of the PCR products were cloned into the pMD19-T vector (TaKaRa, Japan) for sequencing using an Applied Biosystems (ABI) 3730 DNASequencer. The full-length cDNA sequences were obtained by overlaying the three sequences of cDNA.

The deduced amino acid sequences of *Ch*Lhx9 were compared with previously published sequences of representative invertebrate and vertebrate Lhx9s. The sequences were analyzed based on nucleotide and protein databases using BLASTN and BLASTX, respectively^1^. The molecular weight and the theoretical isoelectric point were calculated using the Compute pI/Mw tool^2^. The protein secondary structure and domains were predicted using SMART^3^. Multiple sequence alignments and a phylogenetic tree were constructed using the MEGA7.0 software based on the alignment of the complete amino acid sequences. The cNLS Mapper^4^ was used to predict the nuclear localization signals of *Ch*Lhx9, and Jalview software was used to generate the consensus sequence of the predicted NLS in *Ch*Lhx9 and the proved NLSs.

### Cell Culture and Subcellular Localization

HEK 293T cells were maintained in Minimum Eagle’s medium (Gibco, United States), which contained 10% fetal bovine serum (Gibco, United States) and 1× antibiotics (streptomycin and penicillin, Gibco) at 37°C in a humidified incubator of 5% CO_2_. The ORF of *Ch*Lhx9 was inserted into the pEGFP-N1 vector (Promega, United States) to produce GFP-tagged *Ch*Lhx9 expression plasmids. The recombinant endo-free plasmids, including *Ch*Lhx9-GFP (200 ng) or pEGFP- N1 (200 ng), were transfected into HEK293T cells by using ViaFect Transfection Reagent (Promega, United States). Then, 48 h after transfection, the cells were washed with PBS twice, fixed with 4% paraformaldehyde at room temperature for 15 min, and then stained with DAPI for 5 min. After washing twice with PBS, cellular localization of the *Ch*Lhx9-GFP protein was observed by using fluorescence microscopy.

### Apoptosis Check by Flow Cytometry

Hemocytes were harvested from oysters at 72 h post dsRNA injection. The apoptosis rate was detected by using the Annexin V-FITC detection kit (Vazyme, China) according to the manufacturer’s instruction. In the reaction system, 100 μL of hemocytes, diluted with 100 μL of binding buffer, was incubated with 5 μL of Annexin V-FITC and 5 μL of propidium iodide for 10 min to mark early apoptotic cells and late-apoptotic or necrotic cells. After recollection and re-suspension, the hemocytes were immediately subjected to flow cytometer (Guava, United States) for apoptosis rate detection (10,000 events countered).

### RNA Isolation and Quantitative Real-Time PCR

The total RNA from different tissues and hemocytes of oysters was extracted using TRIzol Reagent (Invitrogen, United States) following the manufacturer’s instructions and then was treated with DNase I (TaKaRa, Japan). We checked the purity of the samples using a Nanodrop Nano-Photometer spectrophotometer (NanoDrop products IMPLEN, United States); the concentration was assessed by Nanodrop2000 (Thermo Fisher Scientific, United States), and the RNA integrity was verified using an Agilent 2100 BioAnalyzer (Agilent, United States).

The cDNA templates for the qRT-PCR were synthesized by the PrimeScript^TM^ RT reagent (with gDNA Eraser) Kit (TaKaRa, Japan). The qRT-PCR reactions were conducted using the 2× RealStar Green Power Mixture Kit (GENE STAR, China) with the LightCycler480II System (Roche, United States), according to the manufacturer’s protocol. Each qRT-PCR analysis was performed in triplicate. The transcript quantifications of the genes were calculated using the 2^-ΔΔC_T_^ method with GAPDH as the reference gene (Kenneth and Livak, 2001).

### RNA-Seq Analysis

Two biological replicates of RNA samples were collected from the *Ch*Lhx9 RNAi or EGFP RNAi group. As previously reported (Mortazavi et al., 2008; Wang et al., 2009), a total of four libraries were constructed, and sequencing was performed with the sequencing platform BGISEQ-500 (BGI, China). Clean tags were generated from raw sequencing reads, which were filtered to remove the adapters, unknown biases and low-quality reads and were mapped to the *C. hongkongensis* transcriptome dataset (Tong et al., 2015). For gene expression analysis, the matched reads were calculated and then normalized to RPKM using RESM software (Li and Dewey, 2011). The significance of the differential gene expression was based on the Noiseq method (Tarazona et al., 2011) with the absolute value of log_2_-Ratio ≥ 1 and the divergence probability ≥ 0.8 in this research. All DEGs were further subjected to Gene Ontology (GO) and KEGG Orthology (KO) enrichment analysis by mapping the DEGs to the GO and KEGG databases followed by hypergeometric tests (Ye et al., 2006; Kanehisa et al., 2008). The reliability of the DEGs was validated by qRT-PCR, and primers (**Supplementary Table S1**) were designed using Primer 5.0 software.

### Statistical Analysis

All of the statistical analyses were carried out with SPSS 18.0 software. Significant differences in the expression of *Ch*Lhx9 in different tissues and in hemocytes after bacterial challenge were analyzed by one-way analysis of variance (ANOVA) followed by Duncan’s multiple range tests. Significant differences in knockdown efficiency of RNAi and flow cytometry analysis of apoptosis were analyzed by Student’s t-test. Means ± SE (standard error) were determined based on three biological replicates.

## Results

### Sequence Analysis of *Ch*Lhx9

**FIGURE 1:**
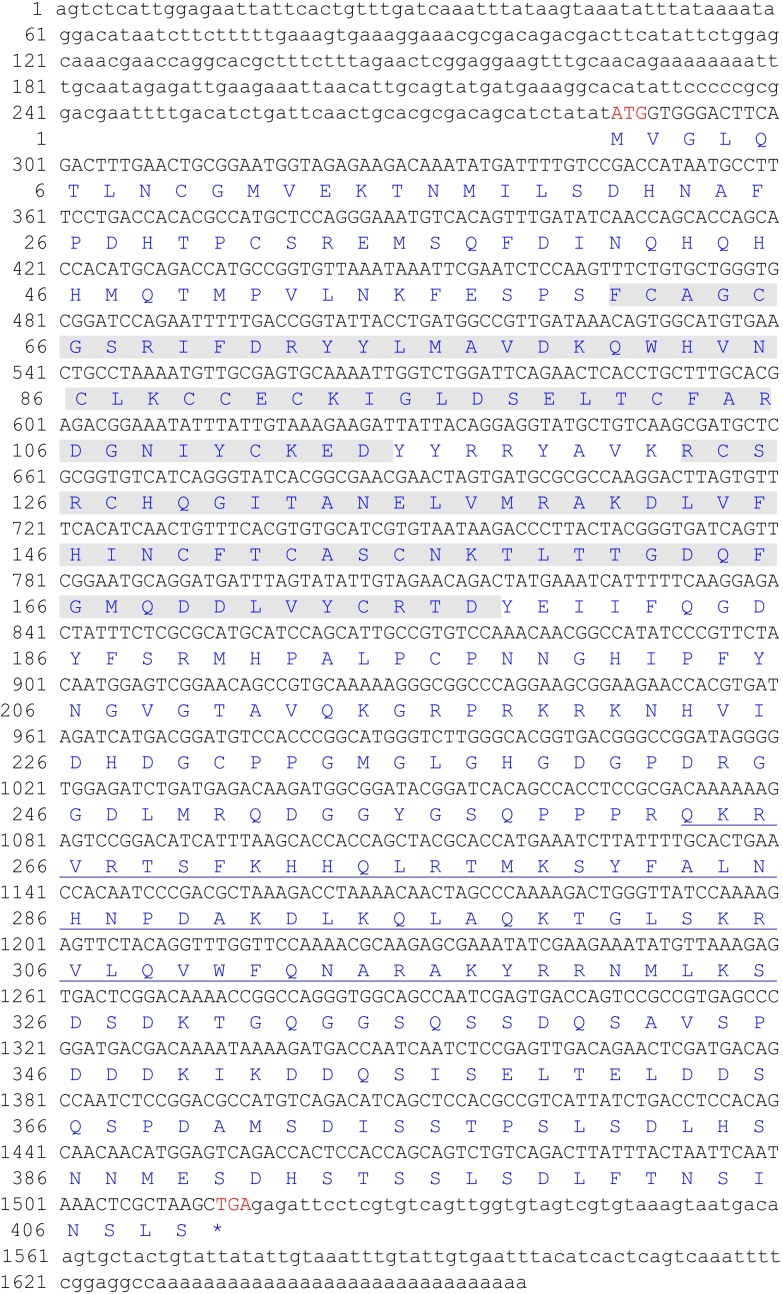
The full-length cDNA and deduced amino acid sequence of *Ch*Lhx9. The two LIM domains (amino acids 61-114 and 123-177) were shadowed and the homeodomain (amino acids 263–325) was underlined.

**FIGURE 2:**
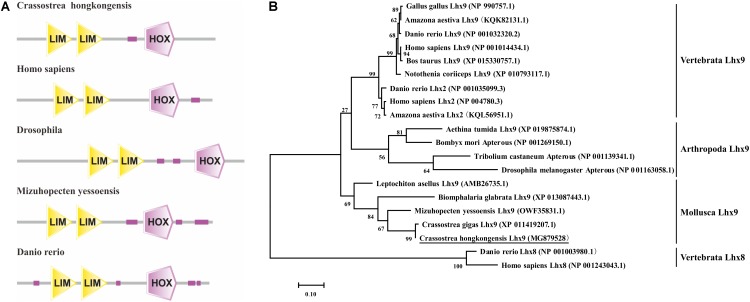
Sequence analysis of Lhx9 proteins. **(A)** Functional domains of different species Lhx9 proteins **(B)** Phylogenetic tree analysis of the amino acid sequences of *Ch*Lhx9 protein and its homologs was constructed by the maximum likelihood method and was bootstrapped 1,000 times using the MEGA 7.0 software.

The full-length cDNA sequence of the *Ch*Lhx9 gene (GenBank accession number: MG879528) contains a 5′-UTR of 286 bp, a 3′-UTR of 143 bp and an open reading frame (ORF) of 1,230 bp. The ORF is predicted to encode a protein of 409 amino acids (**Figure 1**), with a calculated molecular mass of 46.07 kDa and a theoretical isoelectric point of 7.23. The conserved domains of *Ch*Lhx9 were predicted and analyzed by the SMART program, including two LIM domains and one homeodomain, with the typical features of Lhx9 family proteins (**Figure 2A**).

A phylogenetic tree was constructed by MEGA7.0 software using the amino acid sequences of the Lhx9 from *C. hongkongensis* and other species, which reveals their clear evolutionary relationship. As shown in **Figure 2B**, the *Ch*Lhx9 was clustered with Lhx9 from other mollusks such as *C. gigas*, *Mizuhopecten yessoensis*, *Biomphalaria glabrata*, and *Leptochiton asellus* and then clustered with arthropods and vertebrates, which means that its evolution is relatively conservative in mollusks and different from vertebrates and other invertebrates.

### Expression Pattern of *Ch*Lhx9 in Tissues

**FIGURE 3:**
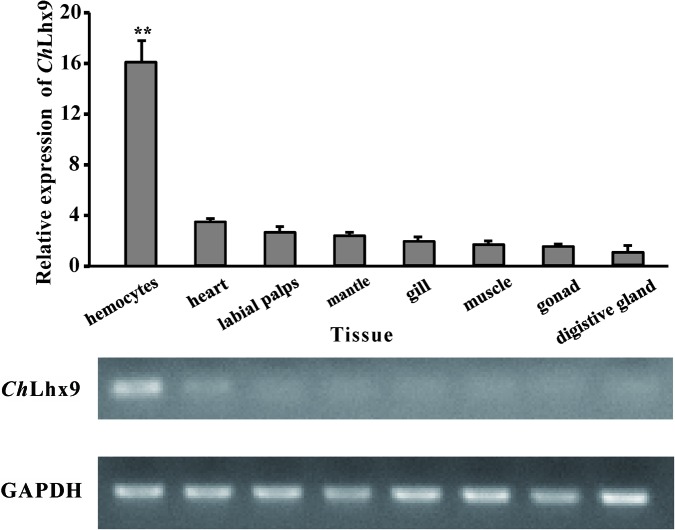
Expression pattern of *Ch*Lhx9. Relative expression levels of *Ch*Lhx9 in different tissues. The Relative expression levels were normalized to GAPDH. ^∗∗^Represent *p* < 0.01.

The mRNA expression pattern of *Ch*Lhx9 was detected via qRT-PCR for all of the examined tissues of *C. hongkongensis*, including the hemocytes, labial palps, heart, digestive gland, mantle, muscle, gonad, and gill. The results revealed that *Ch*Lhx9 was constitutively expressed in all of the examined tissues but had specific and high expression in *C. hongkongensis* hemocytes (**Figure 3**). Its expression level in hemocytes is 4.66-fold higher than in heart and 16-fold higher than in digestive gland.

### *Ch*Lhx9 Localized to the Nucleus

**FIGURE 4:**
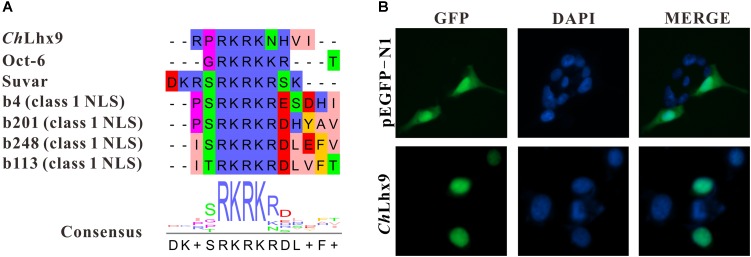
Analysis of subcellular localization of *Ch*Lhx9. **(A)** An alignment showing consensus sequence of predicted NLS in *Ch*Lhx9 and proved NLSs. **(B)** The location of *Ch*Lhx9-EGFP protein are shown by the green fluorescence and nucleus location is indicated by blue DAPI stain in HEK293T cells.

Usually, the function of transcription factors is coupled with their nuclear localization. The NLS (nuclear localization signal) prediction results showed that *Ch*Lhx9 has one NLS (RPRKRKNHVI) that has a conserved alkaline amino acid motif (RKRK) with other proven NLSs (**Figure 4A**). Moreover, the recombinant plasmid that expresses *Ch*Lhx9-EGFP protein was transfected into HEK293T cells, and the fusion protein was visualized by fluorescence microscopy. The results showed that the *Ch*Lhx9-EGFP fusion protein was dominantly distributed in the nucleus, the majority of which overlapped with DAPI staining. As a control, the pEGFP-N1 protein was widely spread over the whole cells (**Figure 4B**).

### Knockdown of *Ch*Lhx9 Results in Declining of the Apoptosis Rate of Hemocytes

**FIGURE 5:**
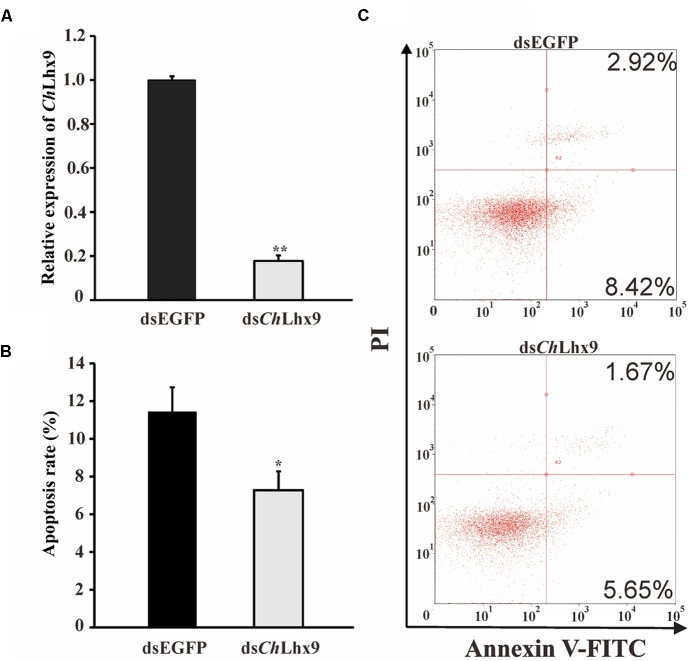
ds*Ch*Lhx9 RNA significantly inhibited apoptosis in *C. hongkongensis* hemocytes. **(A)** Expression levels of *Ch*Lhx9 after RNAi. Significant differences are indicated by an asterisk (^∗^ and ^∗∗^ represent *p* < 0.05 and *p* < 0.01, respectively). **(B)** Histogram showing results obtained from three independent experiments presented **(C)** Representative images of flow cytometry analysis of apoptosis after double staining of *C. hongkongensis* hemocytes with Annexin V-FITC and PI; *n* = 3. Top right region, lower right region and bottom left region represent the late apoptotic cells, early apoptotic cells and living cells respectively.

To investigate the possible function of *Ch*Lhx9 in hemocytes, dsRNA was injected into the muscle to knockdown *Ch*Lhx9 mRNA expression. The results showed that the expression level of *Ch*Lhx9 was successfully knocked down to 19.18% 72 h after the *Ch*Lhx9 dsRNA injection (**Figure 5A**). At the same time, flow cytometry analysis showed that the apoptosis rate of the hemocytes declined 36.49% (including early and late apoptotic cells) in the ds*Ch*Lhx9-injected oysters (**Figures 5B,C**), which clearly implies the pro-apoptotic role of *Ch*Lhx9 in oyster hemocytes.

### Global Expression Profiling of Hemocytes After Knockdown of *Ch*Lhx9

**FIGURE 6:**
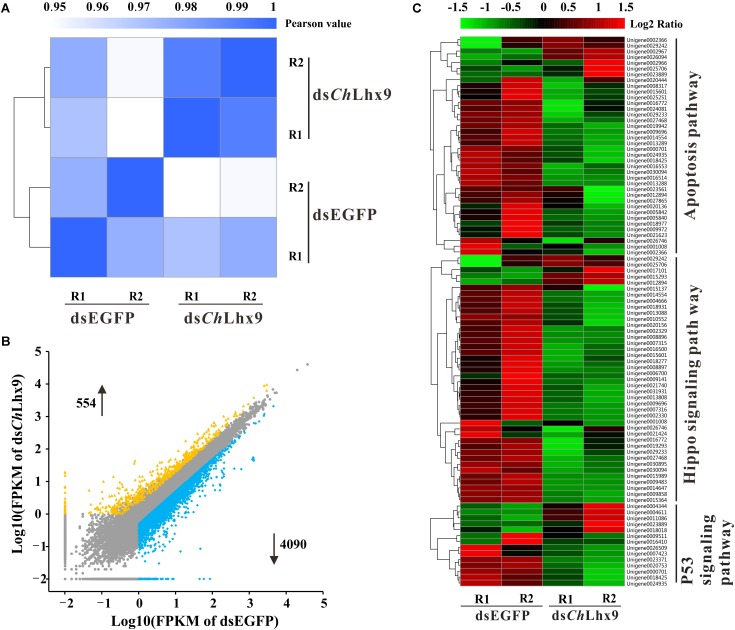
Global expression profile after *Ch*Lhx9 dsRNAi was analyzed by means of RNA-seq quantification. **(A)** Heatmap showing the square of correlation value from two biological replicates in dsEGFP and ds*Ch*Lhx9 groups. The square of correlation value was assessed by using the Pearson correlation. R1 and R2 indicate two independent biological replicates. **(B)** Scatter plot presents the DEGs in hemocytes after *Ch*Lhx9 dsRNAi, DEGs were calculated based on an algorithm of Noiseq, with a cutoff of the absolute log_2_ (RPKM ratio) ≥ 1 and probability ≥ 0.8. **(C)** Three functional gene clusters involved in cell apoptosis were regulated after *Ch*Lhx9 dsRNAi, the color code indicates the fold change of the gene abundance in the form of a logarithm. The RPKM of genes is normalized in each row, and the dendrograms are constructed based on an algorithm of hierarchical clustering.

To study the pro-apoptotic mechanism of *Ch*Lhx9 in hemocytes, we further examined the effect of *Ch*Lhx9 knockdown on the whole transcription expression of *C. hongkongensis* hemocytes. Firstly, two hemocyte libraries, including the *Ch*Lhx9 dsRNA-injected and EGFP dsRNA-injected groups, were generated, and the expression levels of *Ch*Lhx9 in hemocytes of the two replicates was successfully depleted to 27.31% and 25.22% (**Supplementary Figure S1**). We summarized the sequencing and mapping results in **Supplementary Table S2**. RNA-seq analysis also showed that the square of the correlation value is 0.992 and 0.976 between two replicates within the *Ch*Lhx9 dsRNA and EGFP dsRNA injected groups, respectively (**Figure 6A**), which confirms the reliable replication in our assay. In total, more than 73% of the clean reads were aligned to references, and the detailed gene expression is listed in **Supplementary Data Sheet S1**.

Analysis of the differentially expressed genes (DEGs) reveals that 4,092 genes were down-regulated and 554 genes were up-regulated, which suggests an extensive transcriptional activation role of *Ch*Lhx9 in oyster (**Figure 6B** and **Supplementary Data Sheet S2**). Among them, 17 DEGs were selected for qRT-PCR analysis to assess the reliability of RNA-seq, which verifies a high reliability between RNA-seq and qRT-PCR (**Supplementary Figure S2**). Furthermore, pathway enrichment analysis of DEGs showed that the apoptosis pathway, hippo signaling pathway and p53 signaling pathway were significantly enriched after *Ch*Lhx9 knockdown (**Figure 6C** and **Supplementary Data Sheet S2**). Meanwhile, the vast majority of genes of these pathways were downregulated by *Ch*Lhx9 RNAi, which suggests an activating role of *Ch*Lhx9 in controlling the expression of the apoptosis-related pathways.

### *Ch*Lhx9 Induced Apoptosis Through the Transactivation of *Ch*ASPP1

**FIGURE 7:**
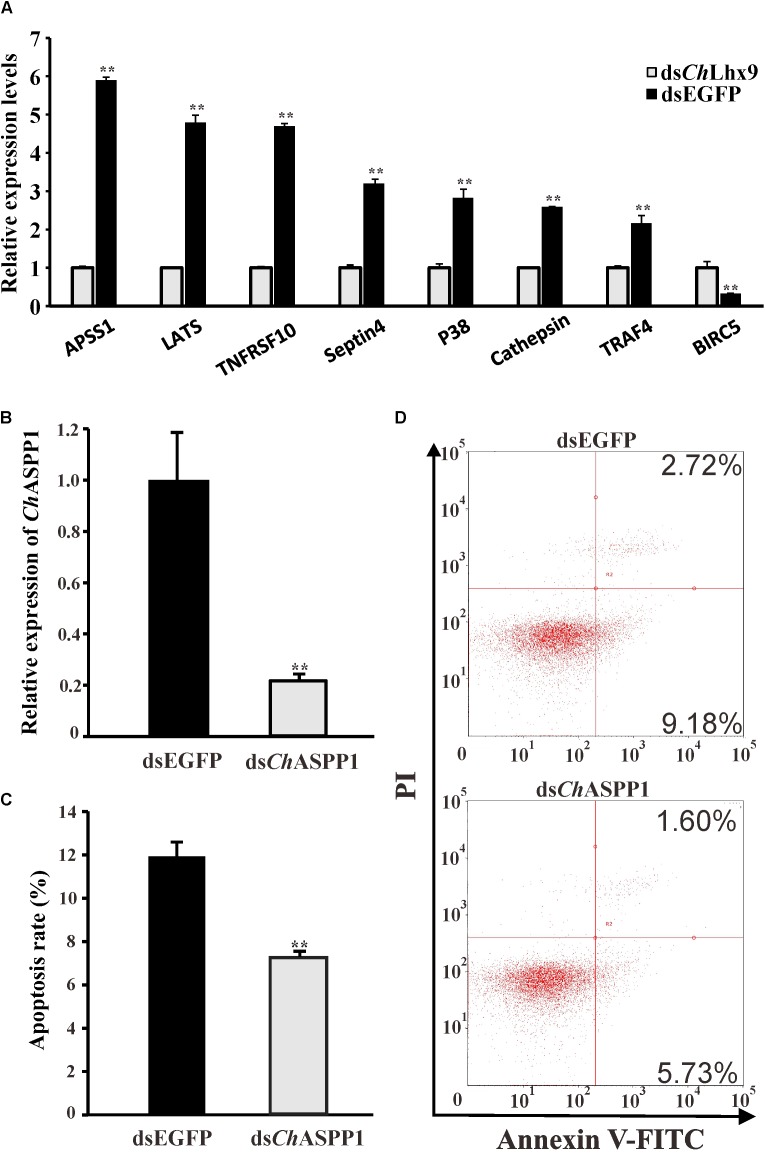
Relative expression levels of selected DEGs and function identification of *Ch*ASPP1. **(A)** Relative expression of apoptosis-related DEGs verified by qRT-PCR. Each qRT-PCR reaction run in three replicates with GAPDH as an internal control. **(B)** Expression levels of *Ch*ASPP1 after RNAi. Significant differences are indicated: ^∗∗^*p* < 0.01. **(C)** Histogram showing results obtained from three independent experiments presented. **(D)** Representative images of fow cytometry analysis of apoptosis after double staining of *C. hongkongensis* hemocytes with Annexin V-FITC and PI; *n* = 3. Top right region, lower right region and bottom left region represent the late apoptotic cells, early apoptotic cells and living cells respectively.

On the basis of several apoptosis-related pathways, the hippo signal pathway, p53 signaling pathway and apoptosis pathway were regulated by *Ch*Lhx9, and the key apoptosis-related genes were further analyzed by qRT-PCR. Among all of the apoptosis-related genes, we found that seven pro-apoptotic and one anti-apoptotic gene had significantly different expression post-*Ch*Lhx9 dsRNA injection, which was tested by qRT-PCR (**Figure 7A**). The expression of ASPP1 (apoptosis-stimulating protein of p53) and LATS1 (large tumor suppressor kinase), pro-apoptotic genes that exist extensively in the model organism, declined to 16.95% and 20.88% of the expression in the control group.

To validate the function of the downstream target genes of *Ch*Lhx9, we injected *Ch*ASPP1 dsRNA into the muscle of oysters. The relative expression of *Ch*ASPP1 was 21.45% of that in the EGFP dsRNA-injected oysters and there was no significant difference in the expression levels of *Ch*Lhx9 between ASPP1-silenced oysters and control oysters (**Figure 7B** and **Supplementary Figure S3**). Compared with the EGFP dsRNA-injected oyster, the hemocyte apoptosis rate of the *Ch*ASPP1 dsRNA-injected oysters declined by 38.92% (**Figures 7C,D**), which verifies that *Ch*ASPP1 was one of the functional targets of *Ch*Lhx9 in the regulation of hemocyte apoptosis.

### *Ch*Lhx9 Regulates Apoptosis in Immune Responses

**FIGURE 8:**
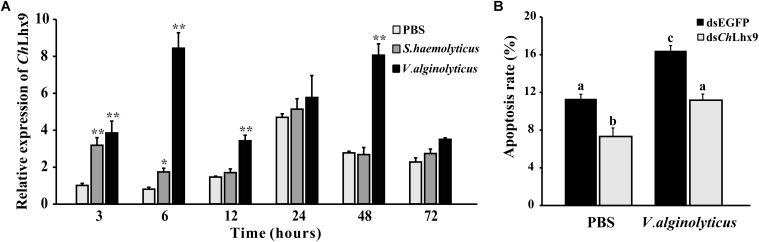
*Ch*Lhx9 involved in immune response and apoptosis check. **(A)** Relative expression levels of *Ch*Lhx9 in hemocytes after by *S. haemolyticus* and *V. alginolyticus* challenge. Each bar represents the mean of the normalized expression levels of the replicates (*N* = 3). Significant differences are indicated by an asterisk (^∗^ and ^∗∗^ represent *p* < 0.05 and *p* < 0.01, respectively). **(B)** Histogram showing results obtained from three independent experiments presented, significant differences are indicated by different letters (*p* < 0.05).

The expression levels of *Ch*Lhx9 in hemocytes were significantly increased in response to two different pathogen challenges. When challenged by *V. alginolyticus*, the expression level initially significantly increased at 3 h post-challenge (3.8-fold), and it reached the highest level at 6 h post-infection (8.4-fold), compared to the treatment with PBS (**Figure 8A**). When challenged by *S. haemolyticus*, the level of *Ch*Lhx9 mRNA was also significantly increased at 3 h and 6 h post-infection, the early stage of infection, and then it recovered to an equal level between the challenge and PBS groups.

Twenty-four hours after the *V. alginolyticus* challenge, the apoptosis rate of the hemocytes was significantly increased with an increased expression of *Ch*Lhx9 (**Figures 8A,B**). The apoptosis rate of hemocytes in the dsEGFP infection group was dramatically increased by 45% compared with the dsEGFP uninfected group. Although the apoptosis rate in the ds*Ch*Lhx9 group was observed to be augmented after *V. alginolyticus* challenge, it still declined 34.48% compared with the dsEGFP infection group.

## Discussion

The LIM homeobox gene Lhx9, which controls the proliferation, differentiation and migration of cells, is essential for organogenesis in vertebrates (the formation of the brain and glands) (Birk et al., 2000; Hobert and Westphal, 2000; Mazaud et al., 2002; Yamazaki et al., 2015; Tandon et al., 2016). The typical Lhx9 protein consists of two LIM domains and one homeodomain that is located C-terminally of the second LIM domain (Hobert and Westphal, 2000), which has the DNA-binding ability to regulate the transcription of downstream genes and is recognized by some co-factors that mediate Lhx9 function (Pomerantz et al., 1995; Jurata and Gill, 1998; Hobert and Westphal, 2000). In this study, the full-length cDNA of *Ch*Lhx9 was cloned from the invertebrate *C. hongkongensis*, which shares conserved domain structure with homologs from other species. Previous studies show that Lhx9 is mainly expressed in the epithelium, developing limbs and the nervous system (Bertuzzi et al., 1999; Mazaud et al., 2002; Tzchori et al., 2009; Yang and Wilson, 2015). However, we found Lhx9 to be specific and highly expressed in hemocytes of oyster (Tong et al., 2015), which demonstrates a species-specific expression pattern during evolution. Thus, it is appealing to explore the possible function of *Ch*Lhx9 in hemocytes and the non-conserved function of Lhx9 in evolution. After the knockdown of *Ch*Lhx9 in oysters, the apoptosis rate of hemocytes declined significantly compared with the control group, which was the first evidence of the pro-apoptosis role of an Lhx9 homolog in oyster. We consider that Lhx9 controls cell proliferation, adhesion and migration in the central nervous system in mouse (Bertuzzi et al., 1999; Rétaux et al., 1999; Tandon et al., 2016). That *Ch*Lhx9 induced the apoptosis of hemocytes in oysters revealed a divergent evolution in Lhx9 function.

To gain comprehensive insight into how *Ch*Lhx9 affects the apoptosis of *C. hongkongensis* hemocytes, global gene expression profiling after *Ch*Lhx9 knockdown revealed that substantial genes were extensively transactivated. More specifically, several crucial pathways that are associated with apoptosis are targets of *Ch*Lhx9, including hippo signaling, p53 signaling and apoptosis pathways. The hippo signaling pathway plays profound roles in both cell density control and anti-tumor because LATS1/2 phosphorylates the transcriptional coactivators, promoting its cytoplasmic localization, preventing the expression of anti-apoptotic genes and inducing apoptosis and restricting cell density (Zhao et al., 2010, 2011). The p53 signaling pathway influences cell cycle arrest, cellular senescence or apoptosis through enhancing or attenuating the functions of the p53 protein (Balint-E and Vousden, 2001; Thorenoor et al., 2016). The tumor suppressor p53 has recently been characterized as an apoptosis-inducing gene in oyster, as it was capable to activate pro-apoptotic genes and induce apoptosis in Pacific oyster, *C. gigas* (Savitskaya and Onishchenko, 2015; Li et al., 2017). Therefore, these evidences revealed that *Ch*Lhx9 can manipulate hemocyte apoptosis through the activation of a variety of apoptotic pathways.

To further define the target genes of *Ch*Lhx9-mediated apoptosis, seven key pro-apoptotic genes, including ASPP1, LATS1, Septin4, TNFRSF10, p38, Cathepsin and TRAF4 and one anti-apoptotic gene, BIRC5, were examined (Xia et al., 1995; Guicciardi et al., 2000; Sax and El-Deiry, 2003; Aylon et al., 2010; Lamers et al., 2011; Shi et al., 2011; Zhao et al., 2014; Zhou et al., 2014; Jeon et al., 2016). The results indicated that seven pro-apoptotic genes were significantly down-regulated and one anti-apoptotic gene was significantly up-regulated in the *Ch*Lhx9 knockdown oysters, which is consistent with the pro-apoptotic role of *Ch*Lhx9. Among them, ASPP1 (apoptosis-stimulating protein of p53) was one of the targets of *Ch*Lhx9 with the highest responsibility in our assay, which has been reported to induce apoptosis by stimulating the selection of proapoptotic genes (Levine, 1997). Additionally, recently, researchers have demonstrated that a blockade of the ASPP-p53 pathway attenuates the apoptosis of retinal ganglion cells (Wilson et al., 2013). In oyster hemocytes, an abatement of the apoptosis rate by knockdown of *Ch*ASPP1 also confirms the conserved function of ASPP1 in apoptosis induction. In addition, LATS1 kinase phosphorylates YAP and TAZ to inhibit cell growth and induce apoptosis (Qi, 2002; Halder et al., 2012), and its homolog, LATS2, phosphorylates ASPP1 and drives its translocation into the nucleus to activate the p53 signaling pathway (Aylon et al., 2010), which implies that its targets could have a synergistic effect on the promotion of apoptosis. However, this system still needs to be investigated further.

Considering the crucial role of hemocytes in immune defense, we believe that *Ch*Lhx9 is involved in the oyster immune system. When challenged with *V. alginolyticus* and *S. haemolyticus*, the expression levels of *Ch*Lhx9 were induced significantly at an early stage of infection. Consistently, the apoptosis rate was also raised under bacterial challenge in oyster, and such a phenomenon was also observed in other marine mollusks (Liu et al., 2017). At the same time, silencing of *Ch*Lhx9 attenuates the apoptosis caused by bacterial infection, which suggests that *Ch*Lhx9 could be an important apoptotic inducer under immune responses. Previous studies have revealed that apoptosis is essential for the development and maintenance of cellular homeostasis of the immune system, to ensure that the host maintains its numbers of immune cells at reasonable levels (Opferman and Korsmeyer, 2003; Hildeman et al., 2007). In addition, the induction of hemocyte apoptosis could prevent autoimmunity and excessive immune response during inflammation (Feig and Peter, 2007). Hence, *Ch*Lhx9-induced apoptosis in hemocytes could play an essential role in homeostasis of the immune responses during infection.

In summary, we first identified a tissue-specific transcription factor Lhx9 homolog (*Ch*Lhx9), which plays a pro-apoptotic role in hemocytes through the transactivation of multiple apoptosis factors or signaling pathways. These results could contribute to a better understanding of hemocyte renewal or homeostasis of immune responses in oyster. However, further studies are required for intensively illustrating a concrete mechanism for how the transcription factor *Ch*Lhx9 regulates the expression of these genes.

## Author Contributions

YiZ, YaZ, and ZY designed the research. YiZ performed all of the experiments with the help of ZH, FM, and JL. SX, YuZ, HM, and ZX cultured and processed the experimental oysters. YiZ and YaZ analyzed the data and drafted the paper. YaZ and ZY critically revised the manuscript and approved the final version to be published.

## Conflict of Interest Statement

The authors declare that the research was conducted in the absence of any commercial or financial relationships that could be construed as a potential conflict of interest.

## Footnotes

1 http://www.ncbi.nlm.nih.gov/BLAST/

2 http://www.expasy.ch/tools/pi_tool.html

3 http://smart.embl-heidelberg.de/

4 http://nls-mapper.iab.keio.ac.jp/cgi-bin/NLS_Mapper_form.cgi\#opennewwindow

## Supplementary Material

FIGURE S1 The knockdown efficiency of ds*Ch*Lhx9 RNAi in RNA-seq. **(A,B)** Represent two biological replicates respectively. Significant differences are indicated: ^∗∗^*p* < 0.01.

FIGURE S2 The correlation coefficient between RNA-seq and qRT-PCR data from 17 genes. The fold change is calculated by gene expression level in dsEGFP group dived by that in ds*Ch*Lhx9 group.

FIGURE S3 The expression level of *Ch*Lhx9 after knockdown of *Ch*ASPP1.

TABLE S1 Primers used in this study.

TABLE S2 Overview of the RNA-seq analysis of *C. hongkongensis* hemocytes: Summary of transcriptome sequencing and mapping results of two replicates are presented in the table.

DATA SHEET S1 List of the FPKM value of *C. hongkongensis* genes.

DATA SHEET S2 List and annotation of differentially expressed genes after ds*Ch*Lhx9 RNAi.
