# Supplementary figures and images for: The Molecular Mechanism Underlying Pro-apoptotic Role of Hemocytes Specific Transcriptional Factor Lhx9 in Crassostrea hongkongensis
Source: Front Physiol. 2018 May 28;9:612. doi: 10.3389/fphys.2018.00612 (PMC5985316; doi:10.3389/fphys.2018.00612)

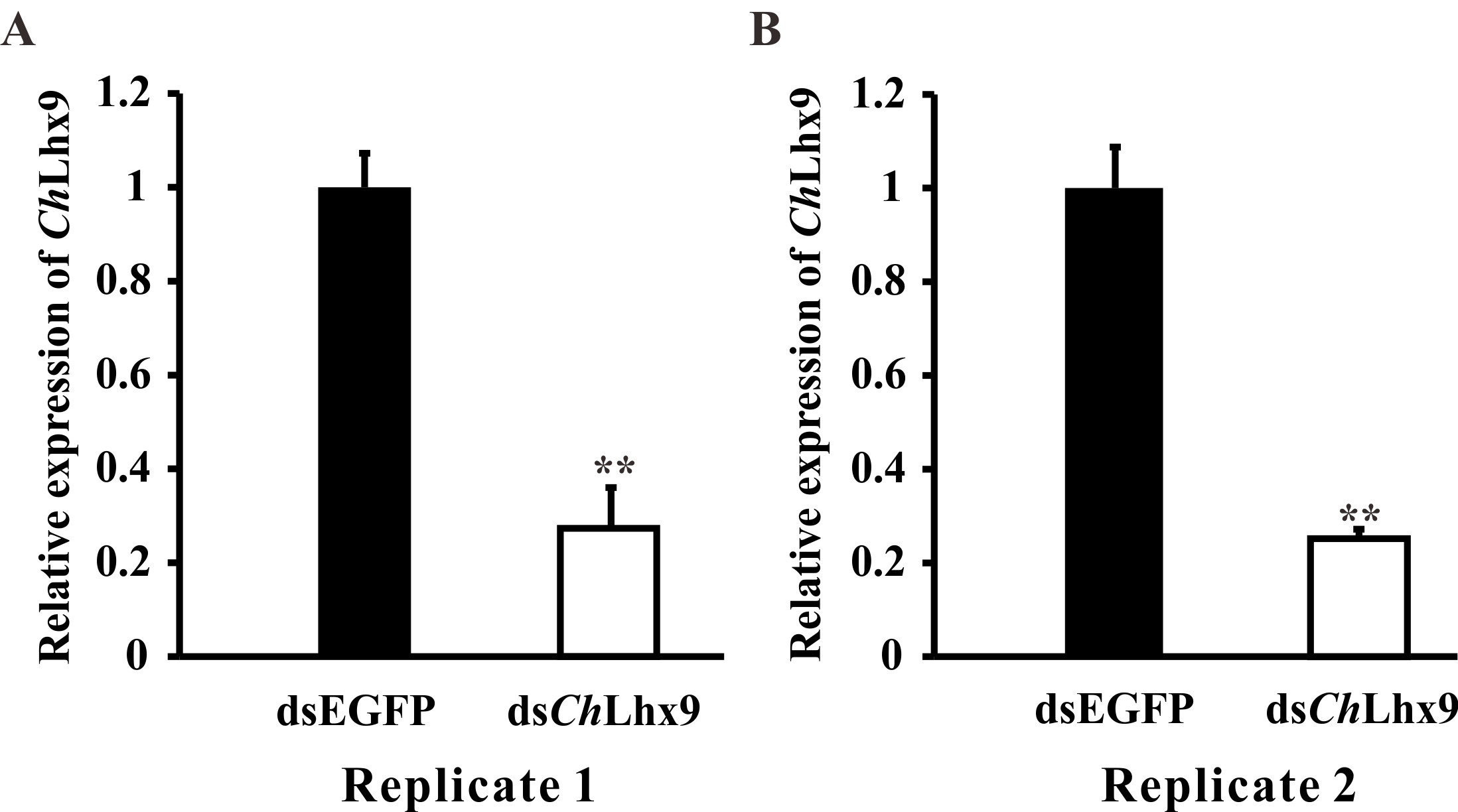

Supplement: FIGURE S1 — The knockdown efficiency of dsChLhx9 RNAi in RNA-seq. (A,B) Represent two biological replicates respectively. Significant differences are indicated: ∗∗p < 0.01. [file Image_1.JPEG]

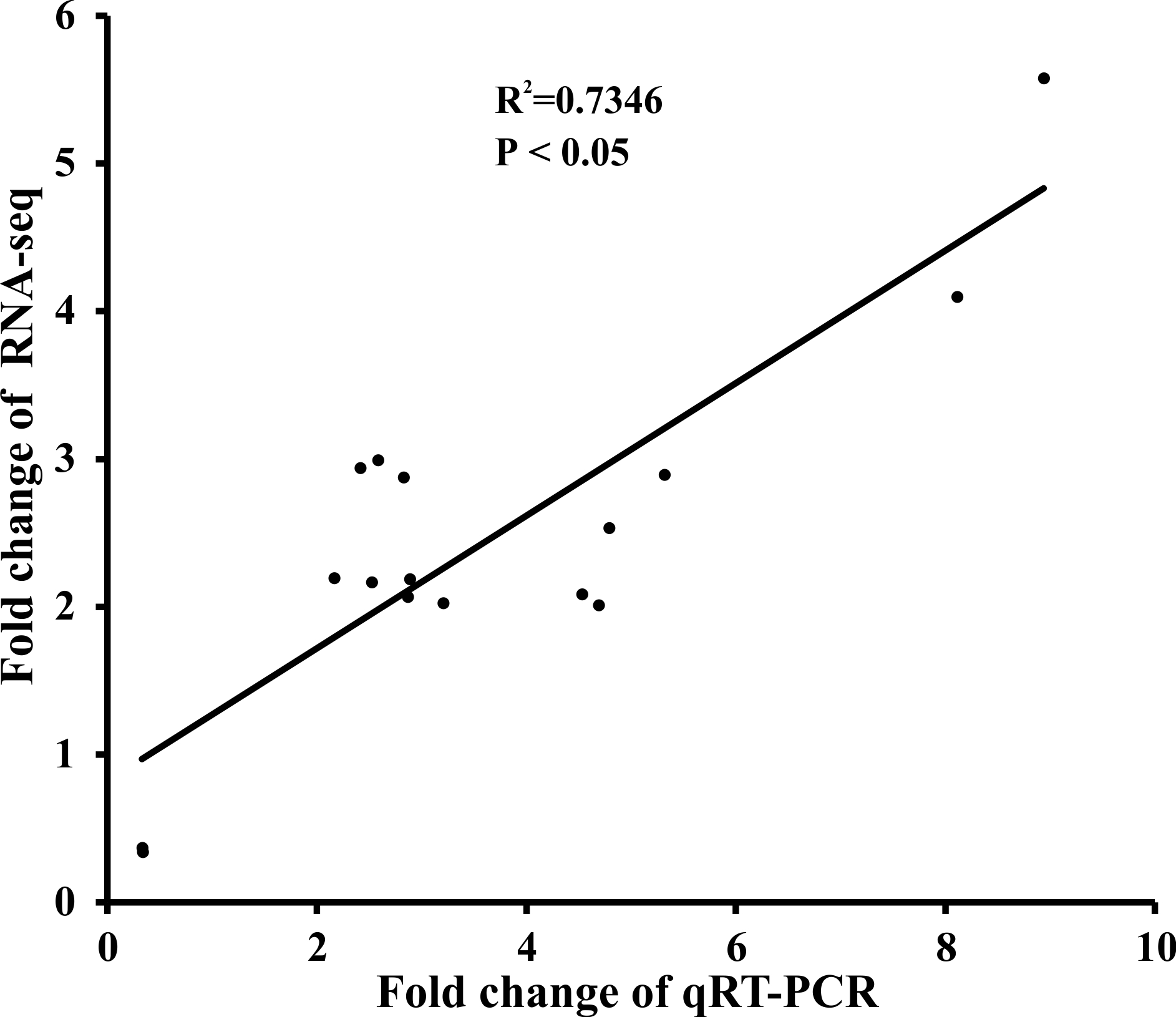

Supplement: FIGURE S2 — The correlation coefficient between RNA-seq and qRT-PCR data from 17 genes. The fold change is calculated by gene expression level in dsEGFP group dived by that in dsChLhx9 group. [file Image_2.JPEG]

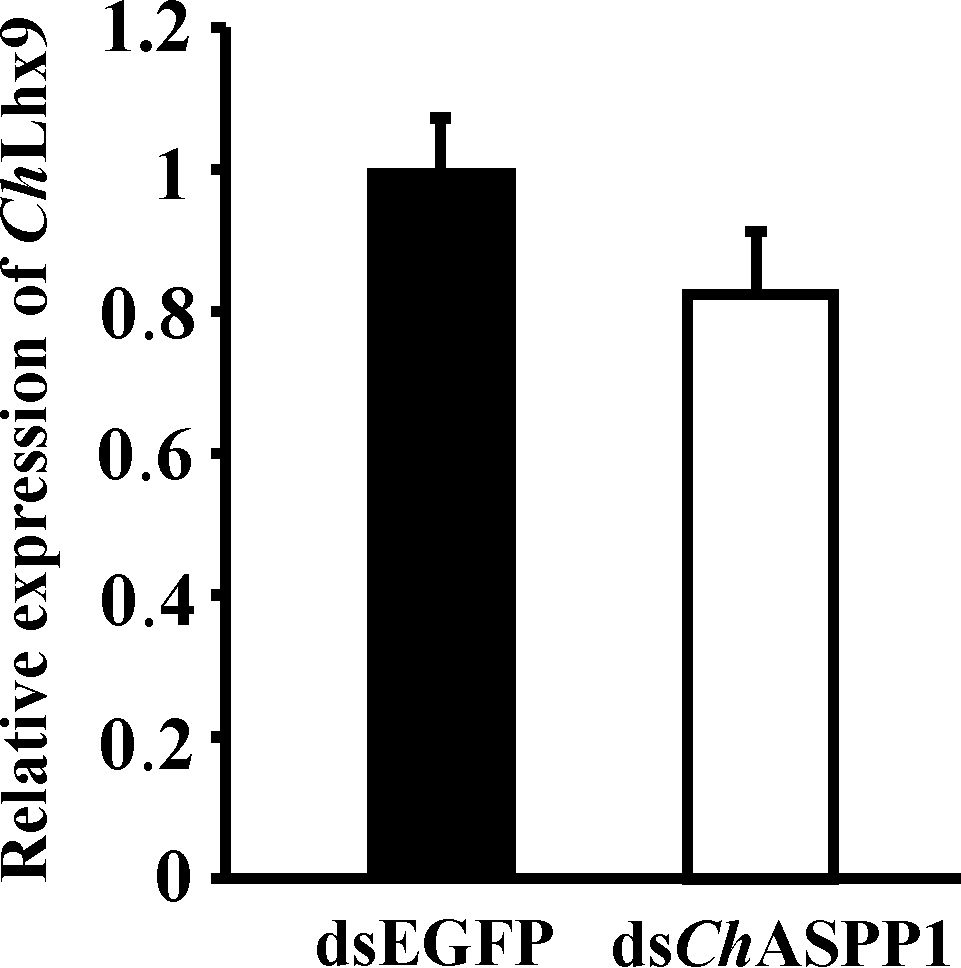

Supplement: FIGURE S3 — The expression level of ChLhx9 after knockdown of ChASPP1. [file Image_3.jpg]
