# Supplementary material for: The Molecular Mechanism Underlying Pro-apoptotic Role of Hemocytes Specific Transcriptional Factor Lhx9 in Crassostrea hongkongensis
Source: Front Physiol. 2018 May 28;9:612. doi: 10.3389/fphys.2018.00612 (PMC5985316; doi:10.3389/fphys.2018.00612)
Supplement: TABLE S1 — Primers used in this study. [file Table_1.DOCX]

**Supplementary Table 1.** Primers used in this study.

| Primer | Sequence (5' to 3') | Comment |
| --- | --- | --- |
| *Ch*Lhx9-F1 | TGGAGCAAGCGAAACAGG | Clone primers |
| *Ch*Lhx9-R1 | CAACTGACACGGGGAATC |  |
| *Ch*Lhx9-F2 | GTCCGACCATAATGCCTTTCC | Real time RT-PCR |
| *Ch*Lhx9-R2 | GATCCGCACCCAGCACAGA |  |
| TakaraUPM | CTAATACGACTCACTATAGGGCAAGCAGTGGTATCAACGCAGAGT | RACE |
| *Ch*Lhx9-F3 | AATCCCGACGCTAAAGACCT | 3’RACE |
| *Ch*Lhx9-R3 | GATCCGCACCCAGCACAGA | 5’RACE |
| GAPDH-F | GGATTGGCGTGGTGGTAGAG | Real time RT-PCR |
| GAPDH-R | GTATGATGCCCCTTTGTTGAGTC |  |
| mTOR-F | GCTTCACTTCATGCTCCACA | Real time RT-PCR |
| mTOR-R | CGGAATCTTCTACCGTCCAA |  |
| LAST1-F | TGCGTCGGATGTTAAATCAA | Real time RT-PCR |
| LAST1-R | CCTTTTCCGCACTAACGAAA |  |
| APSS1-F | TGGAGAGTGTGAAGGCACTG | Real time RT-PCR |
| APSS1-R | ATCGCCTCAAGTTGTCTCGT |  |
| MEF2-F | CCCACCGATAGCTCAAATGT | Real time RT-PCR |
| MEF2-R | ACTAGCTTGCCAGACGCATT |  |
| Titop-F | GCTTGATTTTGGGGCTGTAA | Real time RT-PCR |
| Titop-R | AGGCG TTTATTTTCCGTGTG |  |
| P38-F | CGGAGGAACCTACTGCTGAG | Real time RT-PCR |
| P38-R | TAGGGAGAGGCTGGTTCCTT |  |
| VEGFR2-F | GTCGGTACCAAACCCAAAGA | Real time RT-PCR |
| VEGFR2-R | ATGGCGGGACAAGTTTTATG |  |
| TNFRSF-F | TCTGCAGCTGGGATTTCTTT | Real time RT-PCR |
| TNFRSF-R | CAAGAAAGCAGTGGCAACAA |  |
| TRAF4-F | AGGACCTTGACCTCCATGTG | Real time RT-PCR |
| TRAF4-R | TGCTGTATGCTTCTCCATGC |  |
| GADD4-F | GTCATGACGTCACGTTCCAC | Real time RT-PCR |
| GADD4-R | TTCCTTCCCGCAAATATCAC |  |
| Septin4-F | GATGAAGAAGCTGCACCACA | Real time RT-PCR |
| Septin4-R | GTCACACTCGGGGAACTTGT |  |
| Cathepsin-F | AAACAATGCACCCCTGTAGC | Real time RT-PCR |
| Cathepsin-R | ACAGCTGGAAGGAGGAGTGA |  |
| Calexci-F | CCGGGAACAGACAAGTTCAT | Real time RT-PCR |
| Calexci-R | CCCAAAATCTCCAAGCACAT |  |
| RPTP-F | CCTAGCATCAGCTGGACCTC | Real time RT-PCR |
| RPTP-R | GGATTCTGCACAAACAGCAA |  |
| AAF-F | TGTGTTTTTGGTGGTCCTGA | Real time RT-PCR |
| AAF-R | CGCCTGTCTTCGTAATGGTT |  |
| BRIC5 | TGCATCAGGCTCATCTTCTG | Real time RT-PCR |
| BRIC5 | TGCATCAGGCTCATCTTCTG |  |
| APSS1-RNAi-F | GGATCCTAATACGACTCACTATAGGCTGCGTGGTGAGGTGGAC | Double strand RNA |
| APSS1-RNAi-R | GGATCCTAATACGACTCACTATAGGGGCGAGGATTATGATTGGC |  |
| *Ch*Lhx9-F3 | GGATCCTAATACGACTCACTATAGGCTTGGGCACGGTGACGGGCC | Double strand RNA |
| *Ch*Lhx9-R3 | GGATCCTAATACGACTCACTATAGGGTCAGATAATGACGGCGTGG |  |
| *Ch*Lhx9-F4 | CTACCGGACTCAGATCTCGAGATGGTGGGACTTCAGACTTTGAA | EGFP-N1- ChLhx9 |
| *Ch*Lhx9-R4 | GTACCGTCGACTGCAGAATTCTCAGCTTAGCGAGTTTATTGAATTAGT |  |
| EGFP-RNAi-F | GGATCCTAATACGACTCACTATAGGGATGGTGAGCAAGGGCGAGGA | Double strand RNA |
| EGFP-RNAi-R | GGATCCTAATACGACTCACTATAGGGCTTGTACAGCTCGTCCATGC |  |

“F” indicates forward primers and “R” indicates reverse primers.
