# Supplementary material for: The Molecular Mechanism Underlying Pro-apoptotic Role of Hemocytes Specific Transcriptional Factor Lhx9 in Crassostrea hongkongensis
Source: Front Physiol. 2018 May 28;9:612. doi: 10.3389/fphys.2018.00612 (PMC5985316; doi:10.3389/fphys.2018.00612)
Supplement: TABLE S2 — Overview of the RNA-seq analysis of C. hongkongensis hemocytes: Summary of transcriptome sequencing and mapping results of two replicates are presented in the table. [file Table_2.DOCX]

**Supplementary Table 2.** Overview of the RNA-seq analysis of *C. hongkongensis* hemocytes: Summary of transcriptome sequencing and mapping results of two replicates are presented in the table.

|  | EGFP dsRNA injected group | | *Ch*Lhx9 dsRNA injected group | |
| --- | --- | --- | --- | --- |
|  | 1 | 2 | 1 | 2 |
| Total clean reads | 24,054,265 | 23,527,082 | 23,815,655 | 24,047,172 |
| Total clean nucleotides | 1,202,713,250 | 1,176,354,100 | 1,190,782,750 | 1,202,358,600 |
| Q20 percentage （%） | 97.7 | 97.5 | 97.4 | 97.6 |
| Reads uniquely align to reference gene（%） | 73.9 | 73.09 | 75.41 | 75.11 |
| Total number of unigenes | 29151 | 29816 | 29134 | 29059 |
